# Acute Ocular Pain Following Endoscopic Mastectomy: A Case Report

**DOI:** 10.7759/cureus.88872

**Published:** 2025-07-28

**Authors:** Dongyue Chai, Yanli Zhuo, Lin Lyu

**Affiliations:** 1 Anesthesiology, The Affiliated Hospital of Qingdao University, Qingdao, CHN

**Keywords:** co₂ insufflation, endoscopic mastectomy, intraocular pressure, ocular pain, postoperative complications

## Abstract

Endoscopic breast surgery (EBS) is a widely adopted minimally invasive technique that offers improved cosmetic outcomes and faster recovery. However, the use of carbon dioxide insufflation can occasionally result in unexpected complications. We report a rare case of acute postoperative ocular pain with suspected transient intraocular pressure elevation following EBS in a healthy 36-year-old female. The symptoms were likely due to CO₂ diffusion into subcutaneous tissue and Trendelenburg positioning, which contributed to venous congestion and elevated episcleral venous pressure. Conservative management led to full symptom resolution within 24 hours. This case underscores the importance of intraoperative vigilance and awareness of atypical postoperative symptoms.

## Introduction

Endoscopic breast surgery (EBS) has gained increasing popularity due to its minimally invasive nature, superior cosmetic outcomes, and shorter recovery times, making it a preferred choice for both benign and malignant breast conditions [1]. However, EBS relies on carbon dioxide (CO₂) insufflation to create a retropectoral working space, which can occasionally lead to complications such as subcutaneous emphysema and venous congestion. In rare cases, ocular complications may also arise [2]. While transient increases in intraocular pressure (IOP) have been well-documented in robotic and laparoscopic surgeries - particularly when patients are placed in steep Trendelenburg positions - such occurrences in EBS remain largely underreported [3]. This highlights the need for increased awareness of potential ocular risks associated with EBS, particularly in patients who undergo prolonged or complex procedures.

## Case presentation

A 36-year-old female with left breast malignancy (cT2N1M0, clinical Stage IIB; histologic grade II) underwent endoscopic subcutaneous mastectomy with axillary lymph node dissection and immediate prosthetic reconstruction. She had no previous history of ophthalmic, neurological, cardiovascular, or pulmonary diseases. Preoperative evaluations, including ophthalmologic and imaging studies, were all within normal limits.

The procedure was performed under general anesthesia, and the patient was positioned in a 15° Trendelenburg position. CO₂ insufflation was maintained at 15 mmHg to establish the retropectoral working space. Ocular protection was provided using eyelid patches throughout the surgery. The total surgery duration was 180 minutes, and it was completed without any intraoperative complications.

Postoperative course

Approximately 10 minutes following extubation, the patient immediately reported acute left eye pain (visual analog scale 7/10), along with blurred vision, photophobia, tearing, and a sensation of periorbital pressure. On ophthalmologic examination, mild diffuse conjunctival injection and mild eyelid edema (lid edema) were noted in the left eye. The cornea was clear, and the anterior chamber had a normal depth with no cells or flare. However, IOP in the left eye was elevated at 28 mmHg, while the right eye exhibited an IOP of 16 mmHg, confirming the asymmetry.

Neurological examination showed intact pupillary reflexes, normal extraocular movements, and preserved visual acuity. There were no signs of central nervous system or ocular motor dysfunction. Color vision was tested with the Ishihara plate and was found to be normal.

Diagnostic process

Differential diagnoses considered included acute angle-closure glaucoma: ruled out due to open angles and normal chamber depth; corneal injury or dry eye syndrome: excluded by the absence of epithelial defects and no complaints of dryness or irritation; and increased intracranial pressure (ICP): ruled out as the patient had no headache, vomiting, or papilledema.

The definitive diagnosis was transient elevated IOP likely caused by orbital venous congestion due to CO₂ insufflation and Trendelenburg positioning, as supported by the clinical presentation and IOP measurements.

Treatment and follow-up

The patient was managed conservatively with head elevation to facilitate venous drainage; oral ibuprofen (400 mg every 8 hours) to manage pain and inflammation; topical diclofenac 0.1% (applied twice daily) to reduce ocular inflammation; prophylactic levofloxacin 0.5% (once daily) to prevent potential infection.

These interventions aimed to reduce inflammation and control the elevated IOP. The patient’s symptoms completely resolved within 24 hours.

At one-week follow-up, the patient was asymptomatic, with no recurrence of ocular pain, visual disturbances, or elevated IOP. A thorough ophthalmologic examination confirmed the complete resolution of symptoms, and her visual acuity remained 20/20 bilaterally.

## Discussion

This case highlights a rare but significant complication following endoscopic breast surgery (EBS), particularly in patients undergoing CO₂ insufflation and positioned in Trendelenburg. The patient's ocular symptoms, including acute pain, blurred vision, and elevated intraocular pressure (IOP), were likely caused by a combination of factors. The diffusion of CO₂ into the subcutaneous and fascial planes, particularly in the periorbital region, is a potential mechanism for causing discomfort and pressure in the eye. The CO₂ gas that escapes into these spaces may lead to mild edema and increased episcleral venous pressure, which can transiently raise IOP, resulting in symptoms such as ocular pain and visual disturbances [4, 5].

The Trendelenburg position, used during the procedure to optimize visualization of the surgical area, may also contribute to ocular complications. This position increases venous pressure and impedes venous return from the head, especially when compensatory mechanisms are insufficient. As a result, episcleral venous pressure may be elevated, transiently raising IOP [6, 7]. While this phenomenon is well-documented in other surgical specialties, particularly in laparoscopic and robotic surgeries [8], it is rarely discussed in the context of EBS.

Another consideration is that transient changes in the venous system, especially related to CO₂ insufflation and Trendelenburg positioning, may lead to increased IOP. Given that the patient had no previous ophthalmic or neurological issues, and that other potential causes of ocular symptoms, such as acute angle-closure glaucoma or corneal injury, were ruled out, it seems plausible that the venous congestion associated with CO₂ insufflation and Trendelenburg positioning contributed to the elevated IOP observed [9].

This case underscores the importance for surgeons and anesthesiologists to be aware of the potential for ocular complications in patients undergoing EBS. Although these complications are often transient and resolve with conservative management, early detection and appropriate intervention are essential to prevent prolonged discomfort and to avoid potential damage to ocular structures [10]. Surgeons should be vigilant when managing intraoperative pressures, including minimizing CO₂ insufflation pressure and limiting the duration of Trendelenburg positioning, to reduce the likelihood of such events [11].

Further studies investigating the effects of CO₂ insufflation and Trendelenburg positioning in EBS could provide valuable insights into the pathophysiology and preventative strategies for these rare complications. In addition, real-time monitoring of IOP during long procedures could help detect unusual changes early and mitigate potential risks [12].

## Conclusions

This case demonstrates a rare but significant postoperative complication following endoscopic breast surgery with CO₂ insufflation, where early recognition and conservative management are usually sufficient to resolve ocular symptoms such as acute pain and transient intraocular pressure elevation.
